# The Brooklyn Dental Association

**Published:** 1864-12

**Authors:** Wm. C. Horne


### THE BROOKLYN DENTAL ASSOCIATION.

REPORTED BY DR. WM. C. HORNE.

Sept. 21, 1864.

THE subject for discussion being Dental Instruction :

DR. FITCH remarked that the subject was a most extended one, embracing the whole routine of operative and mechanical dentistry—all of theory and practice. The best instruction is that which is accompanied by demonstration : the old method lacked this. The mallet has introduced an entire change, the pupil being brought to observe all operations.

Much as we may desire to instruct our patients, we must be careful not to expand too much before them, they may listen to all we may say and all the time think it a bore; or that we forget our business. Office instruction is elementary and paramount, but just as necessary is the development and culture derivable from the dental schools : these are highly important. A dental laboratory when well arranged will give instruction in the handling of metals and their preparation ; a fair practical knowledge of chemistry, with the operations in making teeth, etc. But laboratories are generally incomplete, and we must go to the dental college for what they lack. Principles must be inculcated as underlying practice. The dental student should be well grounded in anatomy and physiology.

Dr. Atkinson. Instruction means the forming in the mind of the principles and methods belonging to our necessities. The man so instructed may not be a good practical man. Dentists must be egotistic, but they need not be egotistical; we have to bring out our ideas and put them into sharp definition; subject the hand to the will—express our ideas in the works of our hands. We have too long done mischief by rule, and good by accident, but now we must reverse this: at present we are in a transitional state. By calling out from our own circle expressions of opinion and experience on various points, we will receive floods of light, much more serviceable than what we can attain in months of study: each giving his own light will assist his brother to that which he most needs. Could the present state of dental knowledge be reduced to a permanent form for preservation, it would be a blessing if all the old text books were in ashes.

Dr. Latimer said he had had two preceptors, neither of whom were versed in principles; and who had left him to grope his way just as he could. He would like to see the man who had had proper pains taken with him by his preceptor. They were rare cases. Scientific principles should underlie every man's practice; while the fact was that many practitioners were not as well instructed as they might have been in the public schools. In selecting a pupil it should appear that he had received proper preliminary instruction, and our part should be done faithfully. Dentists should feel an obligation to do their utmost for the education of their pupils, or we shall have unfinished men. No teacher should be above answering the simplest question. We must commence at first principles; the great advantage of a dental college is its facilities for demonstration; mere reading is not sufficient. The student wants to get at what is not in the books. We can not begin too low down and can not aspire too high.

Dr. A. C. Haws said that the most important thing he noticed in his preceptor's operating was, that he never separated teeth where there were two approximal cavities facing each other. In such cases he made one plug answer for both; and if it happened to be of amalgam, the filling was likely to stay in for a long time, much to the satisfaction of the patient, who supposed that every end was answered if it did not come out.

Dr. Marvin was inclined to the old rule to get elementary principles first, and then the powers to be derived therefrom. A college should not receive every applicant, but only such as are qualified; collegiate instruction is necessary to fix scientific knowledge in the pupil's mind. He did not believe in easy learning; nothing is worth acquiring which does not take labor. The young student should have a good substantial education, such as is necessary for an introduction to college; and he should begin his practice under the supervision of a professional man of acknowledged reputation.

Dr. Fitch. It is a common expression that such an one has completed his education; of any one who should appropriate that wise remark he would say, "There is more hope of a fool than of him." We are nothing but students till we go to our graves; a man needs a lifetime to study any branch of science, and he who gets above study retrogrades very fast. We need to keep cramming ourselves with principles or we forget them; we keep our minds bright by constant use. It is extremely difficult to master nomenclature, and the great variety of terms requires constant use to remember them; but it is most necessary to become familiar with these terms. Persons object to having things repeated too often, but it is only in this way that any one can became familiar with this routine. Asking questions is an easy method of getting information; but "easy come, easy go." The best method of learning is by analysis: a man must think for himself—put him on the track, and let him find his own way. Let us be thorough in our investigations, and go as far as any one has ever gone, carrying every thing to its ultimatum. Students are very apt to commit to memory and answer parrot-like; the only way to study anatomy is with the knife; histology, with the microscope; osteology with the bones before you as well as the book: six months can well be devoted to the study of the bones by the student. In conclusion he would say,—never lay down any thing as too hard; where the mind has traveled, there the mind may travel.

